# Bats and ticks: host selection and seasonality of bat-specialist ticks in eastern Europe

**DOI:** 10.1186/s13071-019-3861-5

**Published:** 2019-12-27

**Authors:** Attila D. Sándor, Alexandra Corduneanu, Áron Péter, Andrei Daniel Mihalca, Levente Barti, István Csősz, Krisztina Szőke, Sándor Hornok

**Affiliations:** 1 Department of Parasitology and Parasitic Diseases, University of Agricultural Sciences and Veterinary Medicine of Cluj-Napoca, Cluj-Napoca, Romania; 2 Myotis Bat Conservation Group, Miercurea Ciuc, Romania; 3 Department of Parasitology and Zoology, University of Veterinary Medicine, Budapest, Hungary

**Keywords:** Chiroptera, *Ixodes vespertilionis*, *Ixodes simplex*, *Ixodes ariadnae*, Host–parasite relationships, Underground habitat

## Abstract

**Background:**

Parasites may actively seek for hosts and may use a number of adaptive strategies to promote their reproductive success and host colonization. These strategies will necessarily influence their host specificity and seasonality. Ticks are important ectoparasites of vertebrates, which (in addition to directly affecting their hosts) may transmit a number of pathogens. In Europe, three hard tick species (Ixodidae: *Ixodes ariadnae*, *I. simplex* and *I. vespertilionis*) and at least two soft tick species (Argasidae: *Argas transgariepinus* and *A. vespertilionis*) are specialized for bats.

**Methods:**

Here we report data on the host range of these ticks and the seasonality of tick infestation on wild caught bats in south-east Europe. We collected 1803 ticks from 30 species of bats living in underground shelters (caves and mines) from Romania and Bulgaria. On the basis of tick–host associations, we tested several hypotheses on host–parasite evolutionary adaptations regulating host specificity, seasonality and sympatric speciation.

**Results:**

We observed significant differences in host specificity and seasonality of abundance between the morphologically different bat specialist ticks (*I. simplex* and *I. vespertilionis*) likely caused by their host choice and their respective host-seeking behavior. The two highly generalist, but morphologically similar tick species (*I. ariadnae* and *I. vespertilionis*) showed temporal differences in occurrence and activity, thus exploiting significantly different host communities while occurring in geographical sympatry.

**Conclusions:**

We conclude that bat-specialist ticks show a wide range of adaptations to their hosts, with differences in specificity, seasonality of occurrence, the prevalence and intensity of infestation and all these contribute to a successful division of temporal niches of ticks sharing morphologically similar hosts occurring in geographical sympatry. 
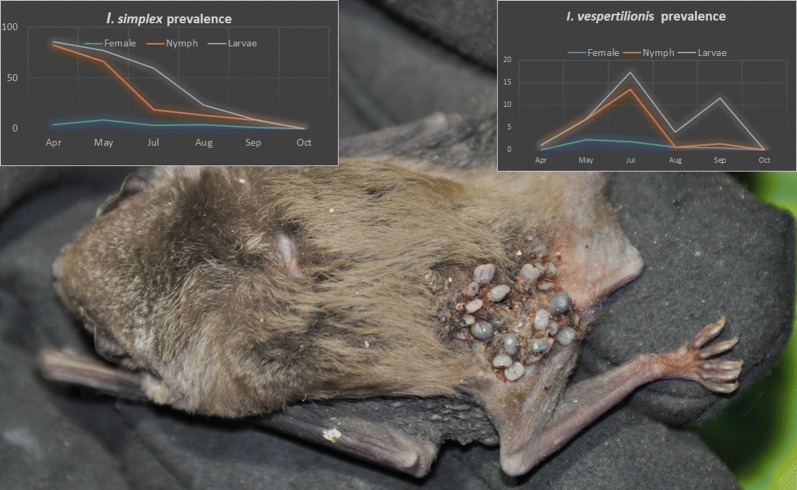

## Background

Most parasite species evolved into organisms highly specialized to their living environment (the hosts’ body) and they also show remarkable behavioral traits helping them to find, colonize and live in/on their host [1]. Parasites may even synchronize their reproduction to access the most profitable host individuals or populations [2–4] or use propagation strategies to increase their chances to find new hosts [5]. Thus, host selection and seasonality of parasites are among the most important ecological aspects of parasite life-cycles to study, and relevant data are crucial to understand associated epidemiological risks.

Ticks (Acari: Ixodidae, Argasidae) are ectoparasites of terrestrial vertebrates [6]. Ticks may show different levels of host specialization, from generalists to the most exclusive species-specific parasites. The majority of them have different groups of vertebrates as hosts at different life stages but some species (especially nest or burrow ticks) are more specific, using a few related species or in extreme cases just one species as a host [7]. From a veterinary-medical point of view, mammals can be regarded as the most important host group, which may be affected by ticks directly (e.g. due to blood loss or inoculation of biologically active compounds), and/or indirectly as a result of infection with tick-borne pathogens.

Ticks of bats (Mammalia: Chiroptera) are usually highly host-specific, i.e. specifically evolved to adapt to their host’s unique morphology and life-style (flight ability, high mobility, the use of underground habitats and thermal tolerance). While there are many studies on the ecology and host–parasite relationships of tick species occurring in Europe, relevant knowledge of bat-specialist ticks is scarce. Most papers studying bat ticks are merely listing incidental host or occurrence records, without data on distribution, seasonality or host choice. Since bats are known as reservoirs for a number of zoonotic pathogens affecting humans and domestic or wild animals, the epidemiological significance of bat-associated tick species has become increasingly recognized. Molecular evidence demonstrated that bat ectoparasites may harbor numerous viruses [8], bacteria [9, 10] or protozoan parasites [11–15] of veterinary and medical importance. These data justify extended research on bat tick ecology and their host–parasite relationships.

European bats are hosts for three hard tick species (Ixodidae: *Ixodes ariadnae*, *I. simplex* and *I. vespertilionis*) and at least two soft tick species (Argasidae: *Argas transgariepinus* and *A. vespertilionis*) [16, 17]. *Ixodes vespertilionis* is a widespread parasite of many bat species of the families Rhinolophidae and Vespertilionidae, commonly occurring in Europe, North Africa and the Middle East [18]. *Ixodes ariadnae* is a recently described species, with rare occurrences in central and western Europe [19]. It is a parasite of bats of the family Vespertilionidae, with nine host species recorded to date [20]. *Ixodes simplex* is a host specialist, primarily a parasite of *Miniopterus schreibersii* and other members of the family Miniopteridae in Europe, North Africa and Asia [18].

These tick species parasitize bats occurring mostly in underground habitats, with their morphology, development and behavior evolved to successfully exploit their hosts in this particular environment. The two soft tick species are primarily parasites of bats using small hollows and crevices for roosting, i.e. chiefly forest bats in Europe [21]. Both species have large distribution ranges, with *A. transgariepinus* occurring in Africa and southern Europe and *A. vespertilionis* in Africa, Asia and Europe [22].

Here we studied the seasonality of occurrence, host choice and temporal distribution of tick developmental stages on bats in central and south-east Europe, focusing on underground-dwelling bats. The aim of this study was to gain information on the host selection and distribution of tick species and developmental stages on different bat species, as well as to test if tick species occurring on bats show reproductive synchronization with their host’s reproduction, as it was shown in a number of other bat–parasite systems [2]. Since ectoparasites with different modes of transmission may respond in different ways to challenges posed by their hosts, we expected differences in infestation patterns between tick species with high host-specialization (*I. simplex*) *vs* those which are more generalists (*A. vespertilionis*, *I. ariadnae* and *I. vespertilionis*).

## Methods

**Fig. 1:**
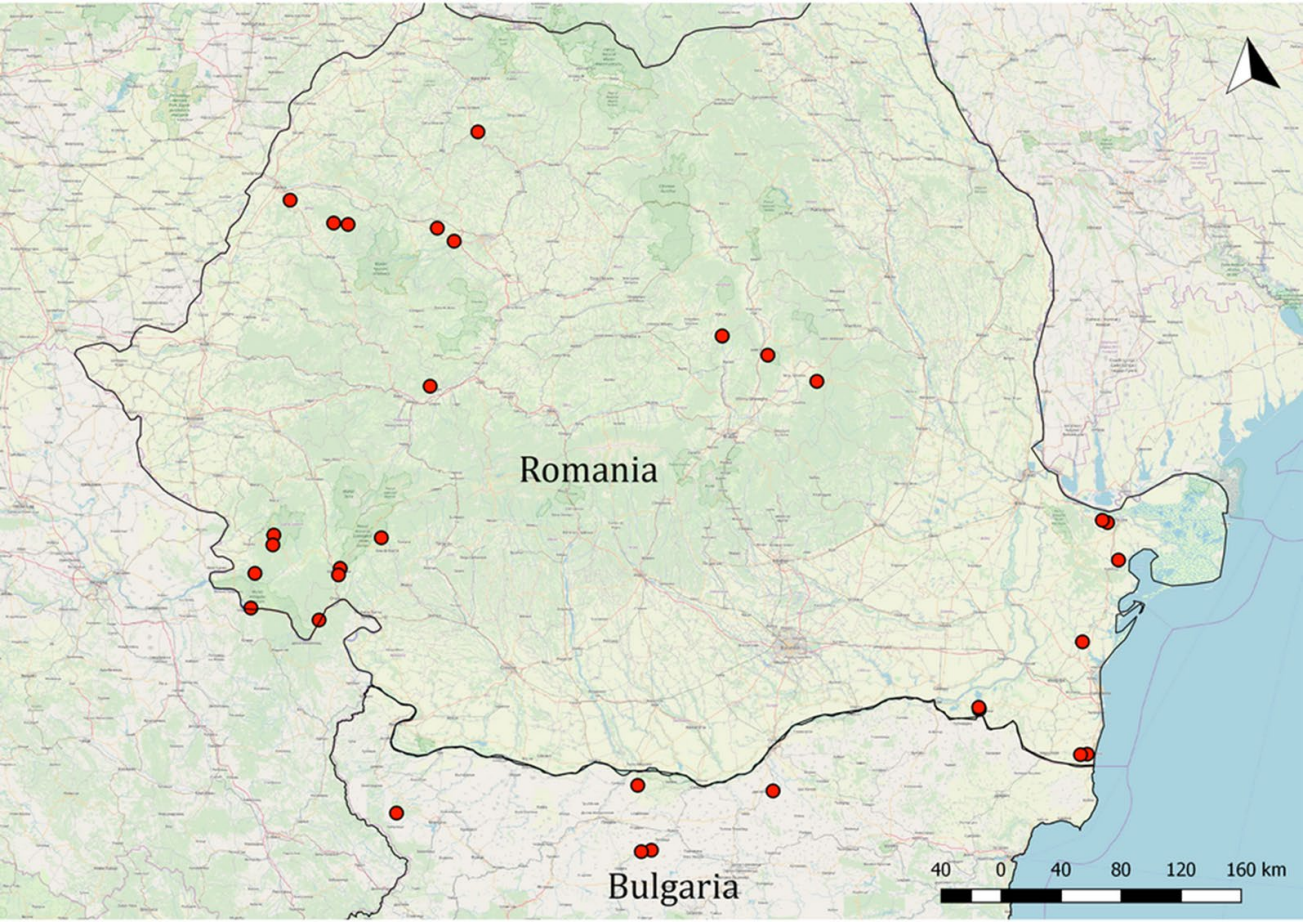
Geographical distribution of sampling locations for bat specialist ticks used in this study

Ticks were collected from live-caught bats. Bats were captured using mist nets and harp traps set close to roost sites, habitat corridors, above small streams and lakes, as well as at swarming sites, in the period 2015–2018 in Romania and Bulgaria (Fig. 1). To limit disturbance, at each site not more than a single one-night capture session was organized in any season. At capture sites we used either a harp trap or one to two monofilament nylon nets or a combination of these. Bat capturing started at sunset and was continuous until bat movement was observable. After removal from the net or the trap, bats were individually stored in cotton bags, until processing. Each bat was identified to species using morphological keys [23], sexed and its age recorded (based on tooth-wear and metacarpal joint ossification). Forearm length and body weight were also recorded for each individual. Before release, bats were marked with a non-toxic dye on the forearm-tip, to avoid pseudo-replication if subsequently recaptured in the same night (the marking fades in a few days). Bats were visually inspected for the presence of ticks by blowing the fur. All ticks were collected using fine forceps and stored in individual, numbered tubes in ethanol. Tick identification was performed under a stereomicroscope using morphological keys [21]. For each tick individual, the development stage and sex (for adults) was recorded.

Bats were grouped into two main groups related to season of capture: all bats captured before mid-May were assigned to the spring group (before reproduction), while bats captured after breeding (in the period of July–October), were assigned to the autumn group (after active nursing and weaning). No bat was captured in the period between mid-May to mid-July to avoid disturbance in the critical period for females and young (pregnancy, and the period of intensive care before weaning) and neither in the hibernating period. For seasonality evaluation we also distinguished early and late parts of spring and autumn. Bat species were also grouped according to their affinity to a particular roost-type in the non-hibernating period to three groups: (i) underground (primarily caves and mines); (ii) building specialists; and (iii) tree specialists [23]. Mean intensity, frequency, prevalence and its 95% confidence interval (CI) were calculated using the software Quantitative Parasitology 3.0 [24] and the statistical software of R [25]. Prevalence and intensity values were compared using Fisher’s exact test and Mann–Whitney U-test, respectively. Differences were considered significant when *P* < 0.05.

## Results

Bats were screened for ticks at 30 geographically different locations in Romania (*n* = 25) and Bulgaria (*n* = 5, Fig. 1). A total of 17 locations were sampled only in one season (5 sites only in spring, 12 sites only in autumn), while the remaining (*n* = 13) were sampled in multiple occasions, more than once in both spring (altogether 35 one-night surveys) and autumn (altogether 38 one-night surveys). As more surveys were completed in autumn, the number of bats captured is higher (2060 bats screened for ticks, 54.9% of all captured bats), with higher diversity of bats sampled (27 *vs* 21 species). However, there were no significant differences in the distribution of bat species holding ticks (Mann–Whitney U-test, *U*_(21)_ = 9.2, *Z* = 2.78, *P* > 0.532), as all but one bat species caught only in autumn (*Myotis brandtii*), had no ticks attached. Altogether 3749 bats were captured, belonging to 30 species (see also Additional file 1: Table S1).

We collected 1803 ticks from a total of 718 bat hosts. Ticks were found on 23 different bat species (Table 1), while the examined individuals of *Barbastella barbastellus*, *Myotis aurascens*, *My. mystacinus*, *Nyctalus lasiopterus*, *N. leisleri* and *Pipistrellus kuhlii* were free of ticks at the time of capture. Tick larvae were the most common stage found on hosts (*n* = 1144; 63.5% of all ticks identified), followed by nymphs (*n* = 597; 33.1%) and females (*n* = 62; 3.4%). No male tick was recorded. The collected ticks belonged to six species and were primarily bat-specialist ticks (*A. vespertilionis*, *I. ariadnae*, *I. simplex* and *I. vespertilionis*), together with two incidental occurrences of generalist tick species (Ixodidae: *Haemaphysalis concinna* and *I. ricinus*). The most common tick species was *I. simplex* (1190 individuals, 66.2% of all ticks), followed by *I. vespertilionis* (319 individuals, 17.7%) and *A. vespertilionis* (279 individuals, 15.5%). *Ixodes ariadnae* was recorded on 10 individuals (0.6% of all ticks) of six bat species (Table 1). Four larvae of *H. concinna* were collected from one *My. mystacinus*, while an *I. ricinus* larva was found on *My. myotis*. These two species have accidental occurrence on bats, thus were excluded from further statistical analyses.

**Table 1:** Bat species captured in eastern Europe, their level of infestation with ticks and the number of ticks collected

**Species**	***n***	**Prevalence (%)** **(95% CI)**	**Intensity** **(95% CI)**	***I. sim***	***I. ves***	***I. ari***	***A. ves***	**Total**
*Miniopterus schreibersii*	1396	37.25 (± 2.55)	2.31 (± 0.16)	1188	12			1200
*Myotis alcathoe*	17	5.88 (± 22.82)	1 (–)			1		1
*Myotis aurascens*	11							
*Myotis bechsteinii*	48	6.25 (± 10.95)	3.33 (± 6.25)		9	1		10
*Myotis blythii*	335	7.76 (± 3.44)	2.77 (± 2.1)		72			72
*Myotis brandtii*	8	25.00 (± 40.1)	2 (–)			2	2	4
*Myotis capaccinii*	194	2.58 (± 3.32)	3.6 (± 6.55)		18			18
*Myotis dasycneme*	3	33.33 (± 57.27)	3 (–)				3	3
*Myotis daubentonii*	302	11.92 (± 4.18)	1.67 (± 0.48)		60			60
*Myotis emarginatus*	80	3.75 (± 6.85)	1.33 (± 1.44)		1	3		4
*Myotis myotis*	149	8.05 (± 5.55)	1.75 (± 0.67)		19	2		21
*Myotis mystacinus*	6							
*Myotis nattereri*	38	10.53 (± 14.27)	2 (± 2.25)		2	1	5	8
*Nyctalus lasiopterus*	1							
*Nyctalus leisleri*	1							
*Nyctalus noctula*	174	3.45 (± 3.95)	6.83 (± 6.72)				41	41
*Pipistrellus kuhlii*	3							
*Pipistrellus nathusii*	19	42.11 (± 24.39)	25.5 (± 26.57)				204	204
*Pipistrellus pipistrellus*	17	5.88 (± 22.82)	9 (–)				9	9
*Pipistrellus pygmaeus*	4							
*Eptesicus serotinus*	29	20.69 (± 19.01)	1.83 (± 1.68)		3		8	11
*Vespertilio murinus*	27	3.70 (± 15.3)	1 (–)				1	1
*Plecotus auritus*	13	7.69 (± 28.31)	2 (–)				2	2
*Plecotus austriacus*	44	4.55 (± 10.95)	2.5 (± 6.08)		1		4	5
*Barbastella barbastellus*	81							
*Rhinolophus blasii*	14	7.14 (± 26.76)	1 (–)		1			1
*Rhinolophus euryale*	189	9.52 (± 5.08)	1.28 (± 0.28)	1	22			23
*Rhinolophus mehelyi*	127	9.45 (± 6.45)	1.58 (± 0.58)	1	18			19
*Rhinolophus ferrumequinum*	307	10.10 (± 3.9)	1.61 (± 0.44)		50			50
*Rhinolophus hipposideros*	135	11.85 (± 6.65)	1.94 (± 1.22)		31			31
Total	3772			1190	319	10	279	1798

*Abbreviations*: CI, confidence interval; n, number of hosts, *I. sim*, *I. simplex, I. ves*, *I. vespertilionis, I. ari*, *I. ariadnae*; *A. ves*, *A. vespertilionis*

Significant differences were detected in prevalence and mean intensity rates of infestation with different tick species. *Ixodes ariadnae* was the rarest bat-specialist tick recorded, followed by *A. vespertilionis* (no differences in prevalence). Significantly higher prevalences were recorded for *I. vespertilionis* (Fisher’s exact test, *P* < 0.001), while *I. simplex* had the highest prevalence, significantly greater even compared to that of *I. vespertilionis* (Fisher’s exact test, *P* < 0.001, see also Table 2).

**Table 2:** Levels of tick parasitism (prevalence and mean intensity) on bats studied in eastern Europe

**Tick species**	**No. of ticks**	**No. of host species**	**No. of infected hosts**	**Prevalence (%)** **(95% CI)**	**Intensity** **(95% CI)**
*Ixodes simplex*	1200	3	518	30.26 (± 2.24)	2.30 (± 0.16)
*Ixodes vespertilionis*	319	15	173	5.11 (± 0.80)	1.84 (± 0.38)
*Ixodes ariadnae*	10	6	7	2.06 (± 2.14)	1.43 (± 0.49)
*Argas vespertilionis*	279	10	26	6.99 (± 3.08)	10.73 (± 8.05)

**Fig. 2:**
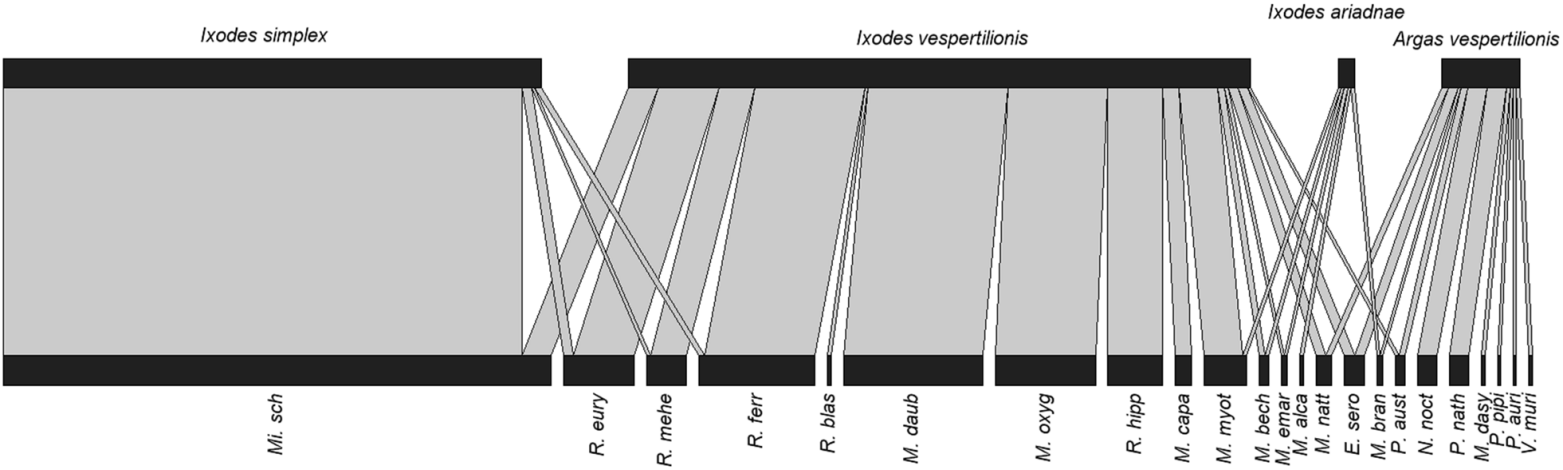
Quantitative interaction web based on bat specialist ticks and their respective bat hosts. Links between nodes represent the sum of individual tick occurrences for a given bat species

The majority of parasitized bats had only one tick (*n* = 346, 47.8%), with the highest count being 88 larvae of *A. vespertilionis* collected from one individual of *Pi. nathusii*. The mean intensity of all *Ixodes* spp. was similar, but significantly higher mean intensity was recorded for *A. vespertilionis* (mean intensity 10.7; Chi-square test, *χ*^2^ = 76.5493, *df* = 1, *P* = 0.0021; Table 2). Tick species differed also in the number of host species used. *Ixodes vespertilionis* exhibited the greatest number of hosts utilized (15 host species), followed by *A. vespertilionis* (10 species), *I. ariadnae* (5 species) and *I. simplex* (4 species, see also Fig. 2). Tick prevalence differed between host species, with overall prevalence ranging between 3.4% and 37.9% (Table 1). Highest overall prevalence was recorded in *Mi. schreibersii* (37.9%), while ticks were rarely found on 7 species (prevalence < 5.0%).

**Fig. 3:**
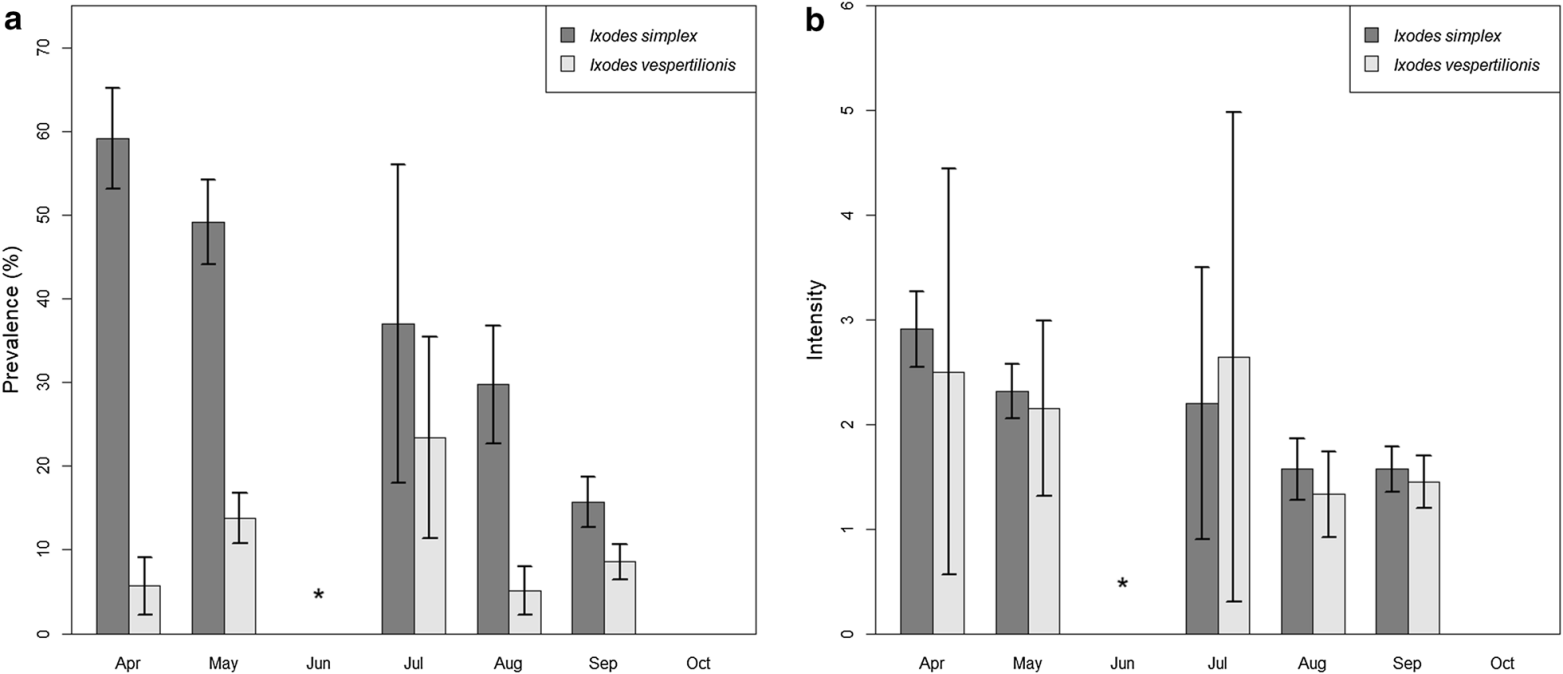
Seasonal differences in prevalence and mean intensity (with standard deviation) of *Ixodes simplex* and *I. vespertilionis* on bat hosts. **a** Monthly distribution of prevalence of tick infestation of bats. **b** Monthly distribution of mean intensity of tick infestation of bats. *, no sampling in June

Tick occurrence showed seasonal fluctuations, with remarkable differences according to tick species. *Argas vespertilionis* was recorded in all sampling months, with most records in the autumn, while *I. ariadnae* was recorded exclusively in the autumn. *Ixodes simplex* was more common in spring and summer (note that no samples were collected during nursing, mid-May to July), decreasing in numbers as the season progressed, with no records in late autumn. This trend was similar for both the overall prevalence and mean intensity rates (Fig. 3). *Ixodes vespertilionis* showed a low prevalence and intensity in the spring and late autumn, with two peaks during summer and early autumn (Fig. 3).

**Fig. 4:**
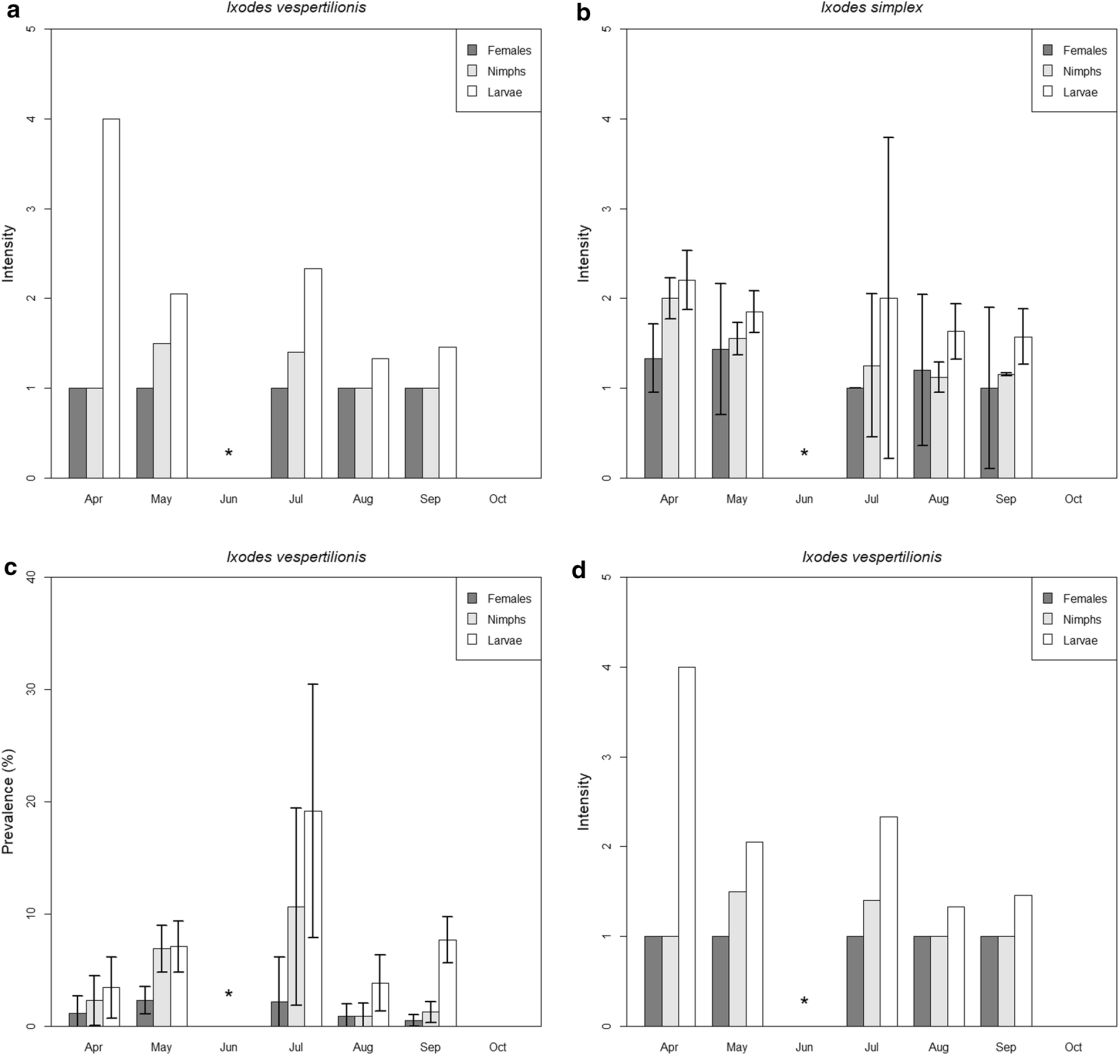
Seasonal trends in the distribution of prevalence and mean intensity (with standard deviation) of different developmental stages of *Ixodes simplex* and *Ixodes vespertilionis* recorded on hosts. **a** Monthly distribution of *I. simplex* prevalence of the different development stages. **b** Monthly distribution of *I. simplex* mean intensity of the different development stages. **c** Monthly distribution of *I. vespertilionis* prevalence of the different development stages. **d** Monthly distribution of *I. vespertilionis* mean intensity of the different development stages. *, no sampling in June

The occurrence of developmental stages varied between different tick species. For *A. vespertilionis* only larvae were recorded, without a clear seasonal trend. For *I. ariadnae*, both larvae and nymphs were observed, but only in the autumn (August–October). Larvae and nymphs of *I. simplex* showed the highest prevalence and intensity in the spring, both decreasing until late autumn. However, the larvae showed a second peak in late July (Fig. 4a, b). Adults of *I. simplex* had low prevalence and intensity and showed little variation in both indices. Larvae of *I. vespertilionis* showed two visible peaks in seasonal prevalence and mean intensity (July and September), while nymphs had only one major peak both in prevalence (July) and mean intensity (May). The females of *I. vespertilionis* showed a higher prevalence in May and July, but both the prevalence and mean intensity rates were low during the entire studied period (Fig. 4c, d).

Host sex also influenced tick distribution. We recorded a significantly higher overall prevalence of ticks on female bats in comparison with males (Chi-square test, *χ*^2^ = 3.9087, *df* = 1, *P* < 0.018); however, no differences were detected for intensity (2.43 *vs* 2.41). This difference could only be demonstrated for cumulative numbers of ticks and hosts, but not if analyzed at the level of individual tick species. For *A. vespertilionis*, the prevalence of ticks on male bats was significantly higher than that on female bats (8.1 *vs* 1.5, Chi-square test, *χ*^2^ = 12.4758, *df* = 1, *P* = 0.0059), while there were no differences in prevalence between the host sexes for *I. ariadnae*. Prevalence of *I. simplex* was significantly higher among female hosts, than among males (46.4 *vs* 31.9%, Chi-square test, *χ*^2^ = 30.008, *df* = 1, *P* < 0.0001). Females also had higher intensities of infestation compared to males (2.4 *vs* 1.8, Mann–Whitney U-test, *U*_(391)_ = 48783, *Z* = 2.165, *P* = 0.030). However, this difference disappeared after the nursing period: during the autumn males and females had nearly similar tick prevalence (28.8 *vs* 25.3%) and mean intensity levels (1.3 *vs* 1.4). No significant differences were found for prevalence and mean intensity related to host sex for *I. vespertilionis*, although males had somewhat higher prevalence and intensity values in both seasons.

## Discussion

### Host selection of bat specialist ticks

In order to have a representative set of data on the host selection of bat specialist ticks, the vast majority of bat species occurring in the south-eastern part of Europe (30 out of the occurring 32 species) were screened during the present study. However, for some of the species, only few individuals could be captured and examined. Taken together, 1803 ticks were collected and evaluated for their host choice, distribution of life stages and seasonality of parasitism. Among the most common bat ticks living in underground shelters, the decreasing order of individual number was: *I. simplex* (*n* = 1190), followed by *I. vespertilionis* (*n* = 319), and the less specialist soft-tick *A. vespertilionis* (*n* = 279). We also managed to collect individuals from the recently described third hard tick species of bats, i.e. *I. ariadnae* (*n* = 10), which usually occurs in a much lower abundance. We recorded the highest rate of parasitism on bent-wing bats (*Mi. schreibersii*, which was also the most common host species in our study with 1396 captured individuals), more than one third of them having ticks (prevalence of 37.25%) with a mean intensity of 2.3. Out of the 1200 ticks collected from this species, 99% were *I. simplex* (showing very high host specificity, Fig. 2), while we also collected 12 individuals of *I. vespertilionis* from this host species (Table 1). The rest of the studied bat species of the families of Rhinolophidae (5 species) and Vespertilionidae (17 species) shared 307 individuals of the far more generalist tick species *I. vespertilionis* (with altogether 10 individuals of *I. simplex* collected from these bat species). Here we report the first occurrence of *I. ariadnae* in Romania and six new host–parasite associations for Romania. We also collected six larvae and four nymphs from seven individual bats captured at two separate and distant locations (in the central part of Romania and in south-east along the border of Romania and Bulgaria). *Ixodes ariadnae* is the rarest bat specialist tick species in Europe, parasitizing mostly *Myotis* bats; to date, nine bat species have been reported as hosts [19, 26–30]. Here we report a new host species (*My. brandtii*) for this tick species. *Argas vespertilionis* is a less specific parasite of bats occasionally infecting other mammals like domestic animals or humans [31, 32] in buildings and, less specifically, in other non-underground shelters of bats (trees, bridges, rock fissures).

The subdivision of bat species as hosts of the two most common bat specialist hard ticks may be explained by the long coevolutionary relationships with their preferred hosts and their different strategies of host-seeking behavior. Ticks feed for a longer period (several days) and they need to fight the host’s immune system and physical defense (preening). Hence, most tick species develop certain adaptations to decrease the above-mentioned defensive strategies. By regulating their immune compatibility to their host’s immune defense system (i.e. increasing their toleration specific to a single host’s immune reaction) they may increase their chances of successful feeding on the selected host species [33]. The social system of the hosts, with special emphasis here on the roosting habits, affects the parasitism of bats [4]. While bent-wing bats (*Mi. schreibersii*) roost in dense groups/colonies with hundreds to thousands of individuals, most vesper- (Vespertilionidae) and horse-shoe bats (Rhinolophidae) rarely form large aggregations (with rare exceptions of females of certain species during pregnancy and nursing [23]). As a consequence, host-seeking behavior of the two different bat specialist tick species should also differ. By specializing to a single host species, *I. simplex* needs to locate the single, but large aggregation of bent-wing bats inside an underground shelter. Although it has adapted to the needs of a single host species (thus decreasing its chances to use other host species), it is more common (in terms of abundance) on its hosts, than *I. vespertilionis* on any other bat species. In contrast, while the more generalist *I. vespertilionis* may use any available bat host for feeding, due to the more scattered spatial distribution of its individual hosts has to seek on a much larger area with a smaller chance of success. This may be the underlying cause also for the morphological differences between the two species, i.e. the considerable differences in the length of their legs.

Assessing host specificity (and the number of host–parasite associations) is important because most parasites that have a broad host range tend to be either important as human parasites or may pose high zoonotic risk due to their vectorial capacity for zoonotic pathogens [34]. While the importance of parasite host range is known, knowledge of complete host ranges is constrained by sampling, especially if many host–parasite associations are rare or hosts have a secretive lifestyle [35].

### Seasonal distribution of different tick species on bats

A number of parasites where shown to synchronize their life-cycle with the reproductive cycle of their hosts [36, 37]. In this way, such parasites gain adaptive advantage to maximize their feeding success and therefore their inclusive or exclusive fitness (reproductive success). Most female bats form small or large aggregations during pregnancy and nursing periods and in these periods they are more susceptible for parasites than males because of their enhanced accessibility, decreased immunocompetence (due to the high metabolic burden of pregnancy and lactating) and the ease of inter-individual parasite transmission [2, 38, 39]. The importance of host’s reproduction timing for parasite prevalence was shown for several ectoparasite species in temperate bat species which roost in underground shelters [3, 40].

Our results demonstrate essential differences between the seasonal distribution of *I. simplex* and *I. vespertilionis* (Figs. 1, 2). Nymphs and larvae of *I. simplex* were highly abundant during spring and their prevalence decreased toward autumn whereas the number of females was constantly low during the study period. Our results indicate that female *I. simplex* ticks produce eggs during the winter and early spring period (hatching of larvae takes 6–8 weeks after females finish feeding, unpublished observation of the authors) and their larvae and nymphs are most abundant during spring (thus before pregnancy starts). They are not able to infect their hosts during winter because most bent-wing bats (*Mi. schreibersii*), even if present all year long at a site, do not use the same locations inside caves for hibernation and nursing [23]. Thus, hosts became available in large numbers only when nursing colonies are initiated (early spring). Consequently, highest prevalence and intensity of *I. simplex* on its host were observed in the early spring period (formation of nursing colonies), continuously decreasing afterwards (Fig. 2). On the contrary, the host species of *I. vespertilionis* ticks tend to change their location often (especially males) and the female bat aggregations (during pregnancy and nursing) are the most accessible resources during the year. The number of *I. vespertilionis* (all life stages) showed a continuous increase from the early spring, with an abundance peak observed in the late nursing (early weaning) period (Figs. 3b and 4c) and decreasing after the nursing period because of their hosts’ active roost-switching behavior during the swarming in late summer and autumn [23]. We found no adult males of any tick species on bats, which is in concordance with the habits of males of bat specialist ticks, which do not feed, but quest on cave walls for engorged females [18].

We found no clear trend in the seasonal distribution of the third common bat-specialist tick species (the bat soft-tick, *A. vespertilionis*). While this burrowing soft tick was recorded in each sampling month and was more common in autumn, the higher number of records in this season is likely due to sampling bias, as much more effort was invested in capturing soft-tick’s hosts in autumn. *Ixodes ariadnae* shows an altogether different seasonal distribution, with all recorded ticks collected in autumn. This is in accordance with previous records [20, 27, 30] and may be an adaptation to the behavior of its most common hosts. Of the ten bats species ever recorded as hosts for *I. ariadnae* only one (*My. myotis*) is a large-sized vespertilionid bat, which occurs year-long in subterranean habitats, while all the other species (*B. barbastellus*, *My. alcathoe*, *My. bechsteinii*, *My. brandtii*, *My. dasycneme*, *My. daubentonii*, *My. emarginatus*, *My. nattereri* and *Plecotus auritus*) are small-sized and occur in underground shelters exclusively or at least primarily in the swarming period (boreal autumn) as they use different shelters in the rest of the year (mainly tree holes [23]). We hypothesize that *I. ariadnae* evolved to adapt to the temporary, high bat species diversity and abundance in the swarming period and its activity is synchronized with its hosts during this period. To test this hypothesis periodical searches should be organized in caves where this species was recorded to assess seasonal differences in its host-seeking activity.

Bat specialist ticks show different adaptations to their hosts, in concordance to their primary hosts’ ecology, life history and social organization. These adaptations may be: (i) both morphological (short *vs* longer legs in *I. simplex* and *I. vespertilionis* [41]) and behavioral (seasonality in abundance) or (ii) just behavioral (seasonality in occurrence). As there are no major morphological differences between *I. ariadnae* and *I. vespertilionis* to reduce interspecific competition [28], these two species target different groups of host species in the same geographical location (inside the same underground shelters) with so-called allochronic activity peaks, i.e. showing geographical sympatry, but temporal allopatry in their activity.

## Conclusions

We conclude that bat-specialist ticks show a wide range of adaptations to their hosts, with differences in specificity, seasonality of occurrence, the prevalence and intensity of infestation and all these contribute to a successful division of temporal niches of ticks sharing morphologically similar hosts occurring in geographical sympatry.

## Supplementary information

**Additional file 1: Table S1.** List of bats captured in Romania and Bulgaria, with details on capture date, host age and sex, together with species and development stage of ticks hosted.

## Data Availability

Data supporting the conclusions of this article are included in Additional file 1.

## Abbreviations

CI: confidence interval
USAMV CN: University of Agricultural Sciences and Veterinary Medicine, Cluj Napoca
IACUC: Institutional Animal Care and Use Committee
